# Lifestyle factors and risk of multimorbidity of cancer and cardiometabolic diseases: a multinational cohort study

**DOI:** 10.1186/s12916-019-1474-7

**Published:** 2020-01-10

**Authors:** Heinz Freisling, Vivian Viallon, Hannah Lennon, Vincenzo Bagnardi, Cristian Ricci, Adam S. Butterworth, Michael Sweeting, David Muller, Isabelle Romieu, Pauline Bazelle, Marina Kvaskoff, Patrick Arveux, Gianluca Severi, Christina Bamia, Tilman Kühn, Rudolf Kaaks, Manuela Bergmann, Heiner Boeing, Anne Tjønneland, Anja Olsen, Kim Overvad, Christina C. Dahm, Virginia Menéndez, Antonio Agudo, Maria-Jose Sánchez, Pilar Amiano, Carmen Santiuste, Aurelio Barricarte Gurrea, Tammy Y. N. Tong, Julie A. Schmidt, Ioanna Tzoulaki, Konstantinos K. Tsilidis, Heather Ward, Domenico Palli, Claudia Agnoli, Rosario Tumino, Fulvio Ricceri, Salvatore Panico, H. Susan J. Picavet, Marije Bakker, Evelyn Monninkhof, Peter Nilsson, Jonas Manjer, Olov Rolandsson, Elin Thysell, Elisabete Weiderpass, Mazda Jenab, Elio Riboli, Paolo Vineis, John Danesh, Nick J. Wareham, Marc J. Gunter, Pietro Ferrari

**Affiliations:** 1 Nutritional Methodology and Biostatistics Group, International Agency for Research on Cancer, 150 cours Albert Thomas, 69372, Lyon CEDEX 08, France; 2 Department of Statistics and Quantitative Methods, University of Milan Bicocca, Milan, Italy; 3 Centre of Excellence for Nutrition (CEN), North-West University, Potchefstroom, South Africa; 4 Medical Research Council, British Heart Foundation, Cardiovascular Epidemiology Unit, Department of Public Health and Primary Care, University of Cambridge, Cambridge, UK; 5 Department of Public Health and Primary Care, University of Cambridge, Cambridge, UK; 6 Department Epidemiology and Biostatistics, School of Public Health, Imperial College London, London, UK; 7 Nutritional Epidemiology Group, International Agency for Research on Cancer, Lyon, France; 8 Centre for Research in Epidemiology and Population Health (CESP), Inserm, Facultés de Médecine Universités Paris Sud, UVSQ, Université Paris Saclay, Gustave Roussy, Villejuif, France; 9 Breast and Gynaecologic Cancer Registry of Côte d’Or, Georges François Leclerc Comprehensive Cancer Care Centre, Dijon, France; 10 Molecular and Genetic Epidemiology Unit, Human Genetics Foundation, Torino, Italy; 11 WHO Collaborating Center for Nutrition and Health, Department of Hygiene, Epidemiology and Medical Statistics, School of Medicine, National and Kapodistrian University of Athens, Athens, Greece; 12 Hellenic Health Foundation, Athens, Greece; 13 German Cancer Research Center (DKFZ), Heidelberg, Germany; 14 Department of Epidemiology, German Institute of Human Nutrition Potsdam Rehbrücke, Nuthetal, Germany; 15 Danish Cancer Society Research Center, Copenhagen, Denmark; 16 Department of Public Health, Aarhus University, Aarhus, Denmark; 17 Public Health Directorate, Asturias, Spain; 18 Unit of Nutrition and Cancer, Cancer Epidemiology Research Program, Catalan Institute of Oncology, Bellvitge Institute for Biomedical Research (IDIBELL), L’Hospitalet de Llobregat, Barcelona, Spain; 19 CIBER Epidemiología y Salud Pública (CIBERESP), Madrid, Spain; 20 Andalusian School of Public Health and Instituto de Investigación Biosanitaria de Granada ibs, Servicio Andaluz de Salud/Universidad de Granada, Granada, Spain; 21 Public Health Division of Gipuzkoa, BioDonostia Research Institute, San Sebastian, Spain; 22 Department of Epidemiology, Murcia Regional Health Council, IMIB Arrixaca, Murcia, Spain; 23 Cancer Epidemiology Unit, Nuffield Department of Population Health, University of Oxford, Oxford, UK; 24 MRC PHE Centre for Environment and Health, School of Public Health, Imperial College London, London, UK; 25 Department of Hygiene and Epidemiology, University of Ioannina Medical School, Ioannina, Greece; 26 Cancer Risk Factors and Life-Style Epidemiology Unit, Institute for Cancer Research, Prevention and Clinical Network – ISPRO, Florence, Italy; 27 Epidemiology and Prevention Unit, Fondazione IRCCS Istituto Nazionale dei Tumori, Milan, Italy; 28 Civi M. Arezzo Hospital, ASP Ragusa, Italy; 29 Città della Salute e della Scienza di Torino Hospital, Turin, Italy; 30 Department of Clinical and Experimental Medicine, Federico II University, Naples, Italy; 31 Centre for Nutrition, Prevention and Health Services, National Institute for Public Health and the Environment, Bilthoven, The Netherlands; 32 Julius Center for Health Sciences and Primary Care, University Medical Center Utrecht, Utrecht University, Utrecht, The Netherlands; 33 Public Health and Clinical Medicine, Nutritional Research, Umeå University, and Arctic Research Centre at Umeå University, Umeå, Sweden; 34 Department of Surgery, Skåne University Hospital Malmö, Lund University, Malmö, Sweden; 35 Department of Public Health and Clinical Medicine, Section of Family Medicine, Umeå University, Umeå, Sweden; 36 Department of Medical Biosciences, Pathology, Umeå University, Umeå, Sweden; 37 Director’s Office, International Agency for Research on Cancer, Lyon, France; 38 MRC Epidemiology Unit, School of Clinical Medicine, Institute of Metabolic Science, University of Cambridge, Cambridge Biomedical Campus, Cambridge, UK

**Keywords:** Healthy lifestyle, Obesity, Cancer and cardiometabolic multimorbidity, Cancer, Cardiovascular disease, Diabetes, Prevention

## Abstract

**Background:**

Although lifestyle factors have been studied in relation to individual non-communicable diseases (NCDs), their association with development of a subsequent NCD, defined as multimorbidity, has been scarcely investigated. The aim of this study was to investigate associations between five lifestyle factors and incident multimorbidity of cancer and cardiometabolic diseases.

**Methods:**

In this prospective cohort study, 291,778 participants (64% women) from seven European countries, mostly aged 43 to 58 years and free of cancer, cardiovascular disease (CVD), and type 2 diabetes (T2D) at recruitment, were included. Incident multimorbidity of cancer and cardiometabolic diseases was defined as developing subsequently two diseases including first cancer at any site, CVD, and T2D in an individual. Multi-state modelling based on Cox regression was used to compute hazard ratios (HR) and 95% confidence intervals (95% CI) of developing cancer, CVD, or T2D, and subsequent transitions to multimorbidity, in relation to body mass index (BMI), smoking status, alcohol intake, physical activity, adherence to the Mediterranean diet, and their combination as a healthy lifestyle index (HLI) score. Cumulative incidence functions (CIFs) were estimated to compute 10-year absolute risks for transitions from healthy to cancer at any site, CVD (both fatal and non-fatal), or T2D, and to subsequent multimorbidity after each of the three NCDs.

**Results:**

During a median follow-up of 11 years, 1910 men and 1334 women developed multimorbidity of cancer and cardiometabolic diseases. A higher HLI, reflecting healthy lifestyles, was strongly inversely associated with multimorbidity, with hazard ratios per 3-unit increment of 0.75 (95% CI, 0.71 to 0.81), 0.84 (0.79 to 0.90), and 0.82 (0.77 to 0.88) after cancer, CVD, and T2D, respectively. After T2D, the 10-year absolute risks of multimorbidity were 40% and 25% for men and women, respectively, with unhealthy lifestyle, and 30% and 18% for men and women with healthy lifestyles.

**Conclusion:**

Pre-diagnostic healthy lifestyle behaviours were strongly inversely associated with the risk of cancer and cardiometabolic diseases, and with the prognosis of these diseases by reducing risk of multimorbidity.

## Introduction

Improvements in longevity have increased the likelihood for an individual to develop two or more diseases, a phenomenon commonly referred to as multimorbidity [1, 2]. Cardiovascular diseases (CVD), including heart disease and stroke, type 2 diabetes (T2D), and cancer are particularly relevant as they are the most common non-communicable diseases (NCDs) and represent major causes of morbidity, disability, and impaired quality of life [3]. Owing to improvements in health care, many individuals will survive their first NCD and multimorbidity is becoming the norm for people with chronic disease [4]. In 2016, there was an estimated 15.5 million cancer survivors living in the USA, of whom 60% of survivors aged 85 years and older had at least one comorbid condition prior to cancer and among the top three prevalent comorbid conditions were T2D and CVD [5]. In addition, data from Scotland have revealed that approximately 65% of individuals older than 65 years were multimorbid and more than half of all those with multimorbidity were younger than 65 years [4]. Multimorbidity is now considered a global health care priority [6].

The study of individual diseases dominates medical research, and epidemiological studies have generally investigated the occurrence of single adverse events as an outcome (e.g. incidence of cancer). This approach provides sound evidence for exposure-disease associations and has greatly advanced our understanding of disease aetiology [7]. The 2013–2020 World Health Organization (WHO) Global Action Plan for the Prevention and Control of NCDs aimed to prevent and control major NCDs and their key risk factors including obesity, tobacco use, physical inactivity, harmful alcohol use, and unhealthy diets [8, 9]. However, limited evidence exists on how established risk factors for single NCDs are related to clustering of NCDs within individuals. This evidence has the potential to broaden the scope of public health recommendations encompassing patients affected by chronic conditions, and to inform on combined interventions to prevent multiple NCDs. A recent randomized controlled trial showed that a multi-domain intervention reduced the risk of accumulating new chronic diseases most effectively in participants who already were affected by at least one chronic disorder at baseline [10]. However, few studies to date have investigated the association of lifestyle factors with multimorbidity [11–16], in particular in combination with cancer [11, 14, 15], and disease trajectories have not been studied.

The aim of this study was to investigate associations between five lifestyle factors and risk of cancer-cardiometabolic multimorbidity defined as developing subsequently at least two morbidities including first cancer at any site, cardiovascular diseases (CVD), and type 2 diabetes (T2D) in an individual. We also estimated 10-year absolute risks for these outcomes.

## Methods

### Study population and design

The EPIC study is an ongoing multi-centre cohort that was initiated from 1992 to 2000 in 23 recruitment centres across 10 European countries (Denmark, France, Germany, Greece, Italy, the Netherlands, Norway, Spain, Sweden, and the UK) and was designed to investigate the relationship between nutrition, lifestyle, genetics, and cancer and other chronic diseases [17]. More than 520,000 adults (70% women) mostly aged 35–70 were recruited and have been followed up for cancer events and mortality status. In addition, two nested cohort studies with a focus on T2D and CVD were established which were used for outcome ascertainment in the current study: EPIC-InterAct is a case-cohort study that aimed to investigate the association between genetic and lifestyle factors and incident T2D events ascertained between 1992 and 2007 in all EPIC countries with the exception of Norway and Greece [18]. Similarly, EPIC-CVD is the component of the EPIC study which investigated the aetiology of cardiovascular disease, with a case-cohort study design that assessed incident coronary heart disease (CHD) and stroke events in EPIC from 1992 to 2010 in all EPIC countries except France [19].

### Assessment of lifestyle exposure

Diet, including alcohol intake, was assessed at baseline using validated country-specific or centre-specific dietary questionnaires designed to capture habitual consumption over the preceding year [17]. Information on smoking status and duration, educational attainment, menopausal status (women), and use of hormones in post-menopausal women was obtained using lifestyle questionnaires [17]. Height and weight (self-reported in the Oxford centre, measured elsewhere) were used to compute body mass index (BMI, kg/m^2^) [17]. Physical activity was assessed at baseline using a specific questionnaire about occupational and recreational activity, from which a validated index (Cambridge Index) was computed to capture all physical activity domains [20]. Adherence to a healthy diet was assessed with the modified relative Mediterranean Diet Score (mrMDS), a variation of the original Mediterranean Diet Score replacing olive oil with vegetable oil [21]. The mrMDS is an 18-point linear score that incorporates 9 nutritional components of the Mediterranean diet, including components presumed to be beneficial (vegetables, legumes, fruit and nuts, cereals, fish and seafood, vegetable oil, and moderate alcohol consumption) and potentially harmful (meat and meat products and dairy products). Each mrMDS component was estimated in grams per 1000 kcal to express intake as energy density [21]. All components were divided into country-specific tertiles and scores 0 to 2 were summed up, with increasing scores for healthier diet (range 0–18).

### Healthy lifestyle index

The five lifestyle factors were summarized in a composite healthy lifestyle index (HLI) as described previously [22]. Briefly, categories of each factor were scored from 0 to 4, with higher points indicating a healthier behaviour for, in turn, smoking (never smoked = 4, former smoker = 2, current smoker = 0), alcohol intake (< 6 g/day = 4, 6.0–11.9 g/day = 3, 12.0–24.9 g/day = 2, 24.0–59.9 g/day = 1, ≥ 60 g/day = 0), physical activity (active = 4, moderately active = 3, moderately inactive = 1, inactive = 0), BMI (22–23.9 kg/m^2^ = 4, < 22 = 3, 24–25.9 = 2, 26–29.9 = 1, ≥ 30 = 0), and diet (scoring 4 to 0 points for top to bottom quintile of the mrMDS). The HLI ranged from 0 to 20. We also computed a simplified version of the HLI (sHLI) by giving one point if the healthy definition of a lifestyle factor was met, according to healthy lifestyle recommendations, and 0 otherwise. The scoring of the sHLI is shown in Additional file 1: Table S1.

### Outcome assessment

Cancers at any site (excluding non-melanoma skin cancer) that occurred among the EPIC cohort were assessed by population cancer registries in Denmark, Italy, The Netherlands, Spain, Sweden, and the UK, and by a combination of methods including health insurance records, cancer and pathology registries, and by active follow-up in Germany. Data on cancer incidence were coded according to the International Classification of Diseases for Oncology (ICD-O-3). Data on total mortality was collected at the recruitment centres through mortality registries and used for censoring [17].

Incident T2D cases were ascertained at each participating centre by a combination of self-report, linkage to primary care registers, secondary-care registers, medication use (drug registers), hospital admissions, and mortality data. Incident T2D cases in Denmark and Sweden were not self-reported but were defined according to local and national diabetes and pharmaceutical registries. To increase the specificity of the case definition for centres other than those from Denmark and Sweden, further evidence was sought for all cases with information on incident T2D from fewer than two independent sources at a minimum, including individual medical records review in some centres [18].

Coronary disease endpoints were defined as any CHD, comprised of myocardial infarction (MI) (ICD-10 codes: I21, I22), angina (I20), or other CHD (I23-I25). Cerebrovascular events were ascertained and validated using the same methods as for coronary events and included haemorrhagic stroke (I60-I61), ischemic stroke (I63), unclassified stroke (I64), and other acute cerebrovascular events (I62, I65-69, F01). First non-fatal coronary events were ascertained by different methods depending on the follow-up procedures by centre, using active follow-up through questionnaires or linkage with morbidity and hospital registries, or both [19]. Validation of suspected events was performed on all ascertained case events (Denmark, Germany, Italy, and Spain) or on a subset of events (Sweden, the Netherlands, and UK). Validation was performed by retrieving and assessing medical records or hospital notes, contact with medical professionals, retrieving and assessing death certificates, or verbal autopsy. Angina was not assessed as a first CHD outcome in the Italian EPIC centres of Varese, Torino, and in Germany, Sweden, and Denmark. Each outcome was classified as fatal or non-fatal with the exception of angina, which is never fatal. In an attempt to harmonize the definition of fatal CVD across centres, non-fatal and fatal events occurring within 28 days of each other were considered to be a single fatal event.

In order to harmonize the follow-up time for the three conditions, incident cases of cancer and CVD ascertained after 31 December 2007 were censored. In EPIC-CVD centres with a censor date earlier than 31 December 2007, incident cases of cancer and T2D were censored at that date. After exclusions of subjects from countries not participating in EPIC-CVD or EPIC-InterAct, i.e. France, Greece, and Norway, subjects with prevalent cancer, myocardial infarction and angina, stroke, and T2D at baseline, with missing information on T2D status at baseline, education, smoking, and physical activity, a total of 291,778 study subjects (64% women) were included in the study (Additional file 1: Figure S1).

### Statistical methods

**Fig. 1:**
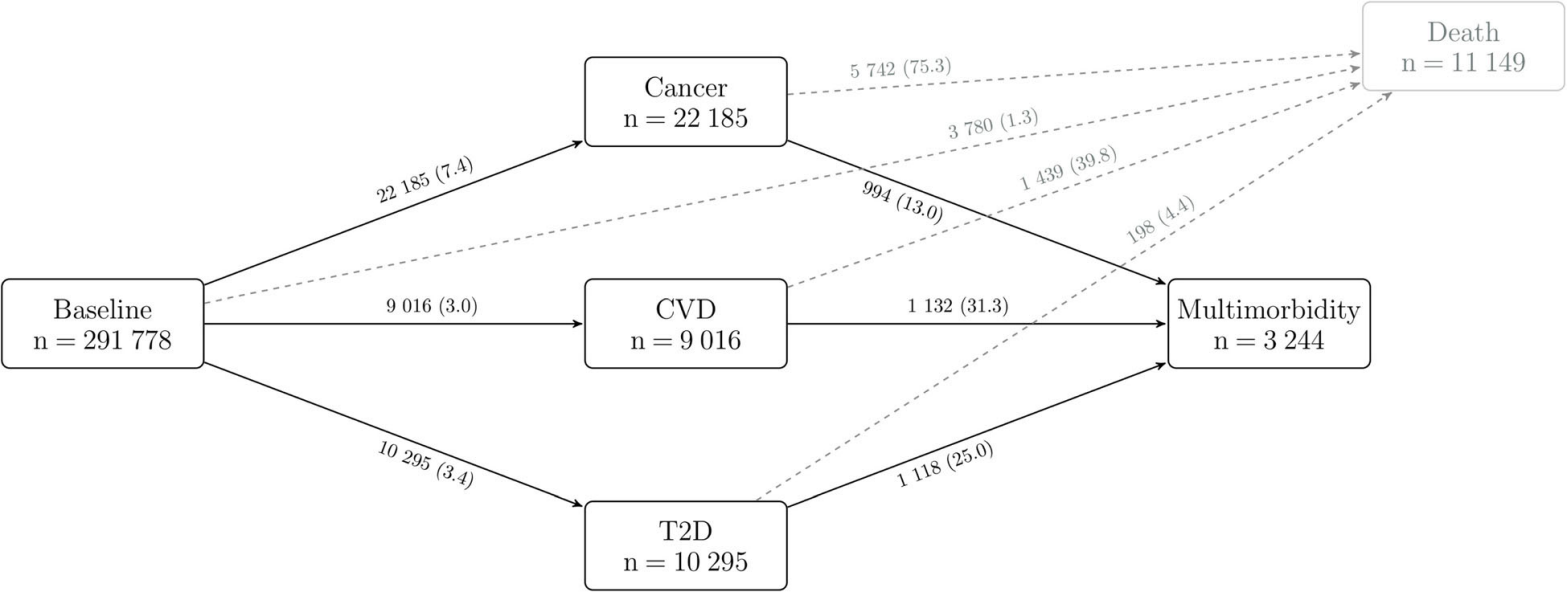
Transitions from baseline to cancer, CVD, T2D, and subsequent cancer-cardiometabolic multimorbidity. Cancer refers to first malignant tumours at any site excl. non-melanoma skin cancer. Deaths were censored and not modelled as a separate outcome. State-specific number of events is reported in boxes, and transition-specific number of events and incidence rates per 1000 person-years (within brackets) are reported on arrows. *CVD* cardiovascular disease, *T2D* type 2 diabetes

Cause-specific hazards for transitions to first conditions, i.e. cancer, CVD, and T2D (including fatal events), and subsequently to multimorbidity were modelled in a multi-state framework with Cox proportional hazard models [23] as outlined in Fig. 1. Deaths were censored and not modelled as a separate outcome. When modelling transitions to first conditions, follow-up was to the date of diagnosis of a first event, 31 December 2007 (or earlier for centres with an earlier censoring date) or the date of death, whichever occurred first. When modelling transitions to multimorbidity, follow-up was considered to be the date of a subsequent second event, 31 December 2007 (or earlier for centres with an earlier censoring date), or the date of death, whichever occurred first.

Cause-specific hazard ratios (HR) and 95% confidence intervals (CI), with age as the primary time variable, were estimated for BMI (continuous, per 5 kg/m^2^), alcohol intake at baseline (continuous, per 12 g/day, non-drinkers were modelled with a separate indicator variable), physical activity (continuous score expressing intensity: inactive, moderately inactive, moderately active, active), smoking status (never [reference], former, current), mrMDS (continuous, per 3-unit increment), and the composite HLI score combining all five lifestyle factors (continuous, per 3-unit increment).

The model was stratified by sex, age at inclusion (1-year categories), recruitment centre, and transitions to allow for transition-specific baseline hazards and estimated transition-specific parameters [24]. Parameter estimates were further adjusted for an indicator variable to define alcohol non-drinkers, education level (no schooling, primary [reference], secondary, and university or more), height (continuous), and energy intake from non-alcohol sources (kcal/day). In women, models were further adjusted for menopausal status (pre-menopausal [reference], peri-menopausal, post-menopausal, surgical) and use of hormones (never [reference], ever, unknown). Analyses were repeated in a priori defined subgroups. Sensitivity analyses were carried out further adjusting for hypertension (yes/no), determined as systolic blood pressure of at least 140 mmHg or diastolic blood pressure of at least 80 mmHg and excluding, in turn, each component of the HLI. In order to evaluate disease trajectories according to the fatality of cancers, we divided total cancer into two groups based on their 5-year survival rates [25]: (a) greater than 40% and (b) below 40%.

Cumulative incidence functions (CIFs) were estimated to compute 10-year absolute risks to develop, in turn, first cancer, CVD (both fatal and non-fatal), T2D, and multimorbidity, according to the disease trajectories described in Fig. 1. Non-fatal CVD events only were considered for the transition from CVD to multimorbidity. Non-Markovian multi-state models that included time since diagnosis for the transitions from any first condition to death or multimorbidity were implemented and CIFs were estimated using the simLexis function of the R package Epi. CIFs were obtained for participants aged 55 and 65 years, separately for men and (post-menopausal) women, comparing healthy (HLI = 15, 85th percentile) and unhealthy (HLI = 5, 4th percentile) lifestyle behaviours. These values correspond, on average, to four and one healthy behaviours on the simplified HLI, respectively. CIFs were averaged over the distribution of the observed adjustment factors (centre, level of education, height, binary indicator for alcohol consumption, total energy intake, and for women, use of hormones).

Competing risks analysis was accounted for in the estimation of cause-specific hazard ratios and of CIFs. In our study, participants at baseline were at risk of developing a first disease and at risk of dying. Similarly, participants who developed a first chronic condition were at risk of developing multimorbidity and at risk of dying.

Statistical tests were two-sided, and *P* values less than 0.05 were considered statistically significant. Statistical analyses were performed with Stata 14.2 (College Station, Texas, USA) and R [26].

## Results

Country- and sex-specific baseline characteristics of the study population are reported in Table 1. Among the 291,778 study participants (64% women), the number of first incident events ascertained within each NCD and after a median follow-up time of 10.7 years (IQR 9.3–12.1) were 22,185 primary cancers (62% in women), 9016 CVD events (42% in women), and 10,295 T2D events (50% in women) (Fig. 1). After an overall median follow-up time of 11.0 years (IQR 9.8–12.3), 3244 participants (41% women) developed multimorbidity (Fig. 1). The most common multimorbidity pattern was CVD among cancer patients with a crude incidence rate equal to 16.6 events per 1000 person-years. Similarly common were T2D among CVD patients (14.7/1000) or cancer among T2D patients (14.3/1000). Among cancer patients, CVD and T2D were equally common (6.5/1000) (Additional file 1: Figure S2).

**Table 1:** Sex- and country-specific characteristics of study participants

Men	*N*	Cancer*	CVD*	T2D*	Multimorbidity†	Age years	Follow-up‡	§Alcohol g/day	||Alcohol NC, %	BMI kg/m^2^	¶Smokers %	¶mrMDS %	¶Physically active %
Italy	13,158	887	483	438	119	49.9 (7.5)	10.2	25 (0.5–66)	4.0	26.3 (3.3)	31.6	55.5	27.6
Spain	13,730	1367	923	1238	375	50.3 (7.1)	13.4	33 (1.7–89)	13.9	28.4 (3.4)	40.4	66.8	21.8
UK	10,862	632	224	80	42	46.0 (13.7)	11.1	14 (0.3–51)	0.1	24.6 (3.4)	13.8	41.5	24.0
The Netherlands	7073	364	421	123	73	42.7 (11.0)	11.5	20 (0.7–59)	8.7	25.4 (3.5)	38.6	3.4	44.2
Germany	18,612	1445	510	797	158	51.8 (7.5)	9.2	26 (1.5–69)	4.0	26.8 (3.5)	24.6	8.0	23.1
Sweden	19,320	2153	1728	1058	548	50.5 (11.2)	12.0	10 (0.4–32)	9.0	25.5 (3.4)	22.7	2.0	19.2
Denmark	22,459	2435	1546	2058	595	56.4 (4.3)	10.6	29 (2.5–79)	1.6	26.4 (3.5)	37.0	6.0	36.5
Total	105,214	9283	5835	5792	1910	51.0 (9.6)	11.0	23 (0.8–68)	5.6	26.3 (3.6)	29.6	23.2	27.1
Women	*N*	Cancer*	CVD*	T2D*	Multimorbidity†	Age years	Follow-up‡	§Alcohol g/day	||Alcohol NC, %	BMI kg/m^2^	¶Smokers %	¶mrMDS %	¶Physically active %
Italy	29,712	1963	449	736	197	50.5 (8.0)	10.0	11 (0.2–36)	22.1	25.6 (4.2)	26.4	55.5	9.4
Spain	22,997	1445	396	1154	194	47.9 (8.2)	13.3	9 (0.2–28)	52.2	27.9 (4.6)	19.5	57.0	4.3
UK	34,802	2119	252	105	57	43.1 (13.5)	11.1	8 (0.4–30)	0.1	23.5 (3.9)	10.3	52.1	17.9
The Netherlands	23,138	2126	1113	490	212	51.6 (11.3)	11.6	11 (0.3–35)	17.2	25.1 (4.1)	27.1	7.7	39.5
Germany	25,785	1443	222	519	67	48.7 (8.9)	9.0	10 (0.4–34)	4.5	25.4 (4.5)	18.7	13.4	19.2
Sweden	23,849	2266	961	892	282	51.1 (10.8)	11.8	6 (0.2–20)	17.1	24.7 (4.2)	24.0	5.9	17.9
Denmark	26,281	2832	809	1676	385	56.7 (4.4)	10.7	14 (0.8–42)	2.5	25.4 (4.3)	31.2	14.1	32.7
Total	186,564	14,194	4202	5572	1334	49.7 (10.7)	11.1	10 (0.3–34)	15.3	25.3 (4.4)	21.9	31.1	19.8

Numbers are means (SD), unless otherwise stated

* Frequency of total incident events among first cancer at any site (excl. non-melanoma skin cancer), cardiovascular disease (CVD), and type 2 diabetes (T2D)

† Frequency of participants developing at least two conditions among first cancer at any site, CVD, and T2D

‡ Median follow-up time in years

§ Among participants drinking more than 0.1 g/day of alcohol at baseline, medians (5th–95th percentile)

|| NC, non-consumers, i.e. participants drinking less than 0.1 g/day of alcohol at baseline

¶ Proportion of current smokers, of study participants in the top tertile of the modified relative Mediterranean diet score (mrMDS), and of participants who are physically active

### Associations with first NCDs

**Fig. 2:**
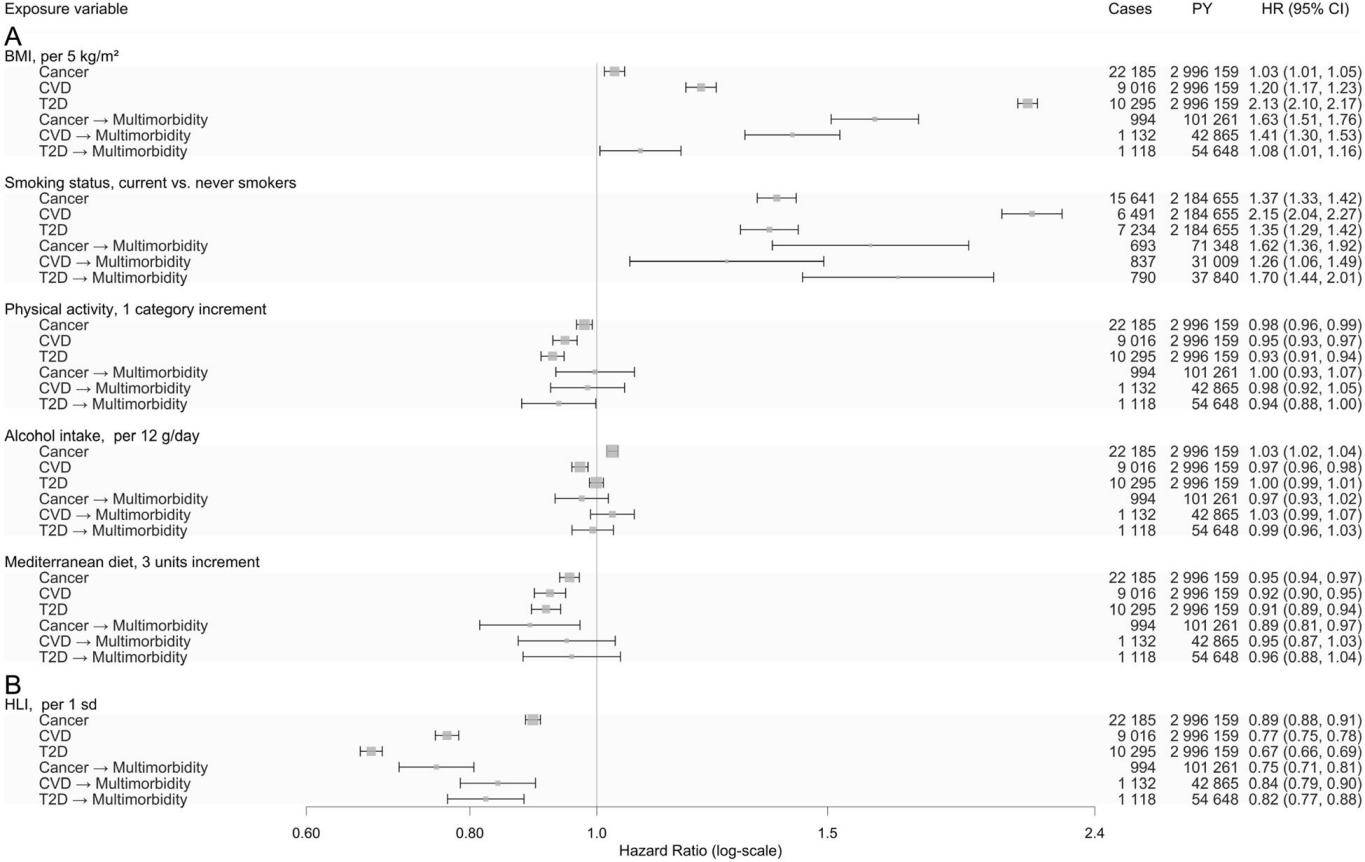
Lifestyle factors associated with risk of cancer, CVD, T2D, and subsequent cancer-cardiometabolic multimorbidity. Cancer refers to first malignant tumours at any site excl. non-melanoma skin cancer. Deaths were censored and not modelled as a separate outcome. **a** Cox proportional hazard models, stratified by age at inclusion (1-year categories), sex, centre, and transition, in a clock-forward multi-state analysis with age as primary time variable, mutually adjusted lifestyle factors and further adjustment for education level (no schooling, primary, secondary, and university or more), height (continuous), an indicator of alcohol use (no/yes), total energy intake (kcal/day), and use of hormones and menopausal status in women. **b** Same as **a**, but the five lifestyle factors were combined in the healthy lifestyle index (HLI); the HLI ranges from 0 to 20 units, with greater scores reflecting healthy lifestyles. *CVD* cardiovascular disease, *T2D* type 2 diabetes

All five lifestyle factors were independently and statistically significantly associated with risk of developing a first NCD with the exception of alcohol consumption, where no association with T2D was observed (Fig. 2). There was a marked heterogeneity in associations, which was most pronounced for body fatness, as indicated by BMI, and smoking status. Body fatness was strongly positively associated with risk of T2D with a HR of 2.13 (95% CI, 2.10 to 2.17) per 5 kg/m^2^ higher BMI, and more weakly with CVD (HR, 1.20 [95% CI, 1.17 to 1.23]) and cancer (HR, 1.03 [95% CI, 1.01 to 1.05]) (Fig. 2). Current smoking, as compared with never smoking, was strongly positively associated with risk of CVD (HR, 2.15 [95% CI, 2.04 to 2.27]), and somewhat weaker with cancer and T2D with HRs of 1.37 (95% CI, 1.33 to 1.42) and 1.35 (95% CI, 1.29 to 1.42), respectively. There was less heterogeneity for physical activity, alcohol intake, and adherence to the Mediterranean diet (Fig. 2).

The HLI was strongly inversely associated with risk of CVD (HR, 0.77 [95% CI, 0.75 to 0.78] per 3 units) and T2D (HR, 0.67 [95% CI, 0.66 to 0.69]), but less strongly associated with risk of cancer (HR, 0.89 [95% CI, 0.88 to 0.91]) (Fig. 2).

### Associations with multimorbidity of cancer and cardiometabolic diseases

Associations between lifestyle factors and transitions to multimorbidity of cancer and cardiometabolic diseases after having had a first NCD are shown in Fig. 2. Little heterogeneity in these transitions was observed with the exception of multimorbidity related to BMI, where HRs per 5 kg/m^2^ higher BMI varied between 1.08 (95% CI, 1.01 to 1.16) for multimorbidity after T2D to 1.63 (95% CI, 1.51 to 1.76) for multimorbidity after cancer.

Physical activity was inversely associated with multimorbidity after T2D with a HR equal to 0.94 (95% CI, 0.88 to 1.00), but not after cancer or CVD. Alcohol consumption was not associated with multimorbidity after occurrence of a first chronic condition. Adherence to the Mediterranean diet was inversely associated with risk of multimorbidity among subjects who had developed cancer with a HR of 0.89 (95% CI, 0.81 to 0.97) per 3 units higher adherence, but not after CVD or T2D.

The HLI was strongly inversely associated with risk of multimorbidity with little heterogeneity after any of the three NCDs (Fig. 2).

### Ten-year absolute risk for NCDs and multimorbidity

**Fig. 3:**
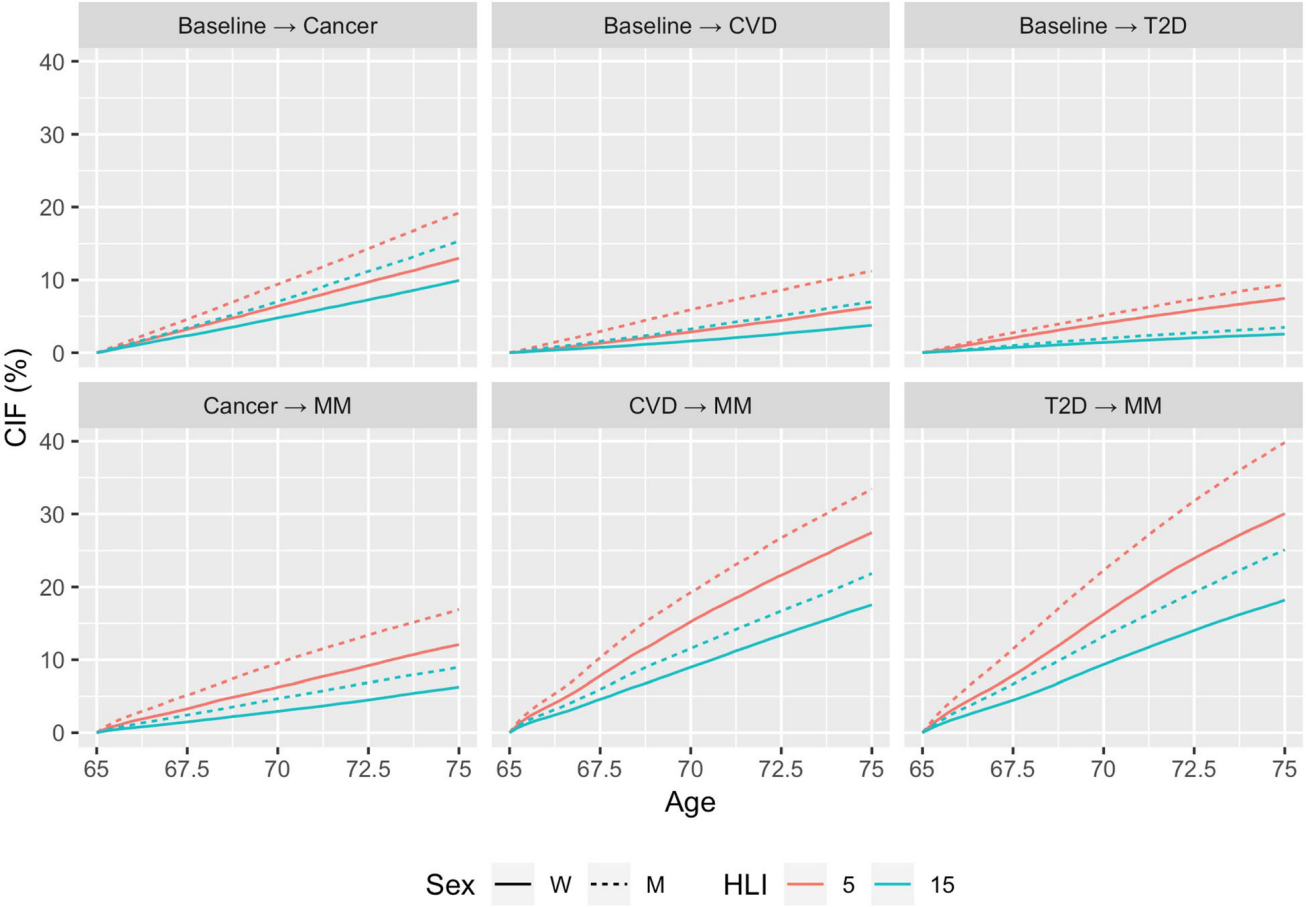
Cumulative incidence functions (CIFs) to develop cancer, CVD, T2D, and subsequent cancer-cardiometabolic multimorbidity. Cancer refers to first malignant tumours at any site excl. non-melanoma skin cancer. Deaths were censored and not modelled as a separate outcome. Computed for 65 years old men (dotted) and women (continuous) for values of the healthy lifestyle index (HLI) of 15 (healthy, 85th percentile in green) and 5 (unhealthy, 4th percentile in red); the HLI ranges from 0 to 20 units, with greater scores reflecting healthy lifestyles. The model was stratified for centre, sex, and adjusted for education level (no schooling, primary, secondary, and university or more), height (continuous), binary indicator of alcohol use (no/yes), total energy intake (kcal/day), and use of hormones and menopausal status in women. *CVD* cardiovascular disease, *T2D* type 2 diabetes

Figure 3 shows the 10-year absolute risks to develop (i) a first NCD, for example, cancer prior to any of the other two conditions (CVD and T2D) accounting for death as a competing event and (ii) subsequent multimorbidity, for men and women at the age of 65 years, for low and high adherence to healthy lifestyles. Despite the increased risk of death among participants, particularly for cancer and CVD patients, risk of developing a second condition was large, in particular for those with poor lifestyle habits. After T2D, the 10-year absolute risks for multimorbidity were 40% and 25% for men and women, respectively, with low adherence to healthy lifestyles (4th percentile of HLI), and were 30% and 18% for men and women, respectively, with healthy lifestyles (85th percentile of HLI). Multimorbidity risks after CVD events were similar, while lower risks were observed in cancer patients. Overall, risks were larger in men than in women.

### Subgroup and sensitivity analyses

Findings were consistent in analyses stratified by sex, age groups (≤ 55 and > 55 years at recruitment), and geographical regions (three groups: Spain, Italy vs. Germany, the Netherlands, the UK vs. Denmark, Sweden) (Additional file 1: Figure S3). The 10-year absolute risks for men and women at the age of 55 years are shown in Additional file 1: Figure S4. Results were only marginally attenuated towards the null after additional adjustment for hypertension and remained statistically significant. Excluding in turn each component of the HLI resulted in similar risk estimates (Additional file 1: Figure S5). Findings were consistent when we used the simplified version of the HLI, which reflects associations per one additional healthy lifestyle behaviour (Additional file 1: Figure S6). Corresponding 10-year absolute risks to develop cancer, CVD, T2D, and subsequent cancer-cardiometabolic multimorbidity for men and women at the age of 65 years for values of the simplified HLI of 4 (adherence to four lifestyle factors) and 1 (adherence to one lifestyle factor) are shown in Additional file 1: Figure S7.

## Discussion

In a large cohort of more than 290,000 adult participants from seven European countries, favourable lifestyle habits, summarized by the HLI score, were strongly inversely associated with incident multimorbidity of cancer and cardiometabolic diseases. Our absolute risk model (CIFs) in particular assessed the burden of multimorbidity among participants who experienced a first disease, and quantified the preventive potential of healthy lifestyle habits with regard to multimorbidity of cancer and cardiometabolic diseases.

The findings of this study are consistent with evidence from investigations that evaluated single NCDs with regard to lifestyle exposures and obesity [9]. Few studies investigated associations between lifestyle factors and risk of multimorbidity [11–16]. In a Finnish population-based cohort of 25–64-year-old men and women (*n* = 32,972), smoking, physical inactivity, and a high BMI were among the main pre-disposing factors of incident multimorbidity defined as the co-occurrence of at least two among T2D, CVD, asthma, cancer, and arthritis [12]. In a small English cohort that included adults aged ≥ 50 years, positive associations with incident multimorbidity, defined as ≥ 2 chronic conditions including T2D, CVDs, cancer, and others, in relation to smoking, higher alcohol consumption, lower physical activity, lower fruit and vegetable intake, and obesity were found [11, 14]. Among 13,714 Australian women aged 45–50 years at enrolment, obesity, hypertension, physical inactivity, smoking, or having other chronic conditions were also significantly associated with increased odds of accumulating cardiometabolic multimorbidity, defined as the co-occurrence of at least two morbid conditions among T2D, coronary heart disease, and stroke [16]. A large pooling study of prospective cohorts from the USA and Europe that included 120,813 adults showed that the risk of cardiometabolic multimorbidity was positively associated with BMI, with a relative risk of 1.9 (95% CI, 1.8 to 2.3) per 5 kg/m^2^ increment [13]. In contrast, lifestyle factors were not associated with 3-year incidence of multimorbidity among community-dwelling older adults 75 years and older [15]. Our findings are largely consistent with previous studies, but also go beyond in that we defined multimorbidity as developing subsequently two incident NCDs. This approach allowed the quantification of the preventive potential with regard to multimorbidity of cancer and cardiometabolic diseases.

The 10-year absolute risk estimates for cancer-cardiometabolic multimorbidity ranged between 5 and 17% for cancer patients, and between 20 and 40% for T2D and CVD patients, depending on sex and adherence to healthy lifestyles. In this respect, it is worthwhile mentioning that once an individual develops a cancer, the competing risk of mortality is comparatively larger than among individuals experiencing T2D or non-fatal CVD. This explains why the absolute risk of developing multimorbidity was lower among cancer patients than among CVD or T2D patients. We further evaluated the risk of multimorbidity in patients developing cancer in sites with 5-year survival below or above 40% [25]. This supplementary analysis indicate that pre-diagnostic healthy lifestyles were particularly beneficial for patients developing cancer with longer survival, where, for example, the 10-year absolute risk of multimorbidity for 65-year old men with healthy and unhealthy lifestyle profiles were 11% and 24%, respectively (Additional file 1: Figure S8). The pronounced sex differences in risk patterns are likely to reflect larger prevalence of key risk factors in men than women. Importantly, the change in absolute risks related to healthy vs. unhealthy lifestyles indicate the preventive potential in terms of risk stratification for multimorbidity of cancer and cardiometabolic diseases. Many modifiable risk factors, including obesity, smoking, and physical inactivity are associated with multiple cancer types, T2D, and CVD, and could be the target of intervention strategies aiming at controlling multiple conditions, beyond efforts focusing on one disease only [10]. A population-based cohort study in the Netherlands showed that participants aged 45 years and older who did not smoke, where of normal weight, and with no hypertension at baseline spent 21.6% of their remaining lifetime with one or more NCDs, compared to 31.8% of their remaining life for participants with all of these risk factors at baseline [27].

Exposure to risk factors that are associated with more than one disease is one plausible explanation for the clustering of NCDs in individuals and indicate common etiological pathways [28, 29]. For example, obesity promotes systemic inflammation, a well-described pathway for the development of cancer [30], CVD [31], and T2D [32].

This study has several strengths. First, we used individual-level data from a large prospective cohort of European adults using validated assessments of cancer, CVD and T2D, together with study participants’ pre-diagnostic exposure to several lifestyle factors. Although EPIC was designed to assess the occurrence of cancer, this study capitalized on the ascertainment of incident CVD and T2D events and related validation efforts carried out within the EPIC-InterAct [18] and EPIC-CVD [19] studies. Second, it is the largest study to date to estimate associations between modifiable exposures and the risk of multimorbidity. Third, associations were modelled in a multi-state framework accounting for the sequence of incident chronic conditions.

The study findings need to be interpreted in light of the following limitations. First, information on lifestyle exposures assessed at baseline was used in this study, while potential changes in modifiable behaviours during lifetime, particularly after the diagnosis of NCDs, could not be accounted for in the investigation. Nevertheless, our results indicate associations between pre-diagnostic lifestyle habits and risk of NCDs and multimorbidity, assuming that exposure profiles prior to disease onset may have an impact on subsequent morbidities. Potential improvements in health behaviours after diagnosis of a first NCD would most likely have led to an underestimation of observed relative and absolute risks. Moreover, it has been shown that in the absence of interventions, the vast majority of individuals do not make major lifestyle changes following diagnosis of a serious chronic disease [33]. Second, associations between risk factors and the risk of multimorbidity after the occurrence of a first disease may be affected by collider bias as exemplified in the obesity paradox [34, 35], and such a bias tends to weaken observed associations. In our study, however, risk estimates were in the expected direction and magnitude after conditioning on the first disease, suggesting that collider bias was of limited concern in our study. An additional limitation was the lack of treatment data after a first NCD. Metformin, which is recommended as first-line therapy in most T2D patients, has been found to decrease cardiovascular risk and possibly some cancers [32, 36]. In contrast, the increased risk of cardiac diseases following cancer therapy is well recognized [37]. However, assuming that treatment is independent of lifestyle, then our observed associations should be unaffected by treatment. Finally, although our relative risk estimates should be generalizable, the absolute risks associated with lifestyle factors heavily depend on the underlying risks of the study population, which however may be different in the general population.

In conclusion, healthy lifestyle behaviours were strongly inversely associated with the risk of cancer and cardiometabolic diseases. Healthy lifestyles prior to a first NCD may also contribute to a favourable prognosis of these diseases by reducing risk of multimorbidity. These findings provide strong support to public health recommendations to adhere to multiple healthy lifestyle factors.

## Supplementary information

**Additional file 1: ** Figure S1. Provides the participant flow chart. Figure S2. Transitions to specific cancer and cardio-metabolic multimorbidity patterns. Figure S3. Results of subgroup analyses. Figure S4. CIFs for men and women of 55 years of age. Figure S5. Results of sensitivity analyses. Figure S6. Results for simplified HLI. Figure S7. CIFs for men and women of 55 years of age using the simplified HLI. and Figure S8. CIFs for men and women of 55 years of age for two groups of cancers. Table S1. Scoring of the healthy lifestyle index (HLI) and its simplified version.

## Data Availability

Study protocol and statistical code: Available from Dr. Freisling (e-mail, freislingh@iarc.fr). Data set: Requests for the data require formal approval by the EPIC principal investigators; for information on how to submit an application for gaining access to EPIC data and/or biospecimens, please follow the instructions at http://epic.iarc.fr/access/index.php.

## Abbreviations

CIFs: Cumulative incidence functions
CVD: Cardiovascular disease
EPIC: European Prospective Investigation into Cancer and nutrition
HLI: Healthy lifestyle index
mrMDS: Modified relative Mediterranean Diet Score
NCDs: Non-communicable diseases
T2D: Type 2 diabetes
