# Supplementary material for: Lifestyle factors and risk of multimorbidity of cancer and cardiometabolic diseases: a multinational cohort study
Source: BMC Med. 2020 Jan 10;18:5. doi: 10.1186/s12916-019-1474-7 (PMC6953215; doi:10.1186/s12916-019-1474-7)

Additional File 1 – BMC Medicine

Lifestyle Factors and Risk of Multimorbidity of Cancer and Cardiometabolic Diseases: A Multinational Cohort Study

Heinz Freisling, PhD*; Vivian Viallon, PhD; Hannah Lennon, PhD *et al.*

*Corresponding Author:

Heinz Freisling, PhD

International Agency for Research on Cancer, World Health Organization

150 cours Albert Thomas

69372 Lyon CEDEX 08, France

Off. +33 472 738 664

Mob. + 33 698 557 239

E-mail: freislingh@iarc.fr

Table S1: Scoring of the healthy lifestyle index (HLI) and its simplified version

Figure S1: Participant flow-chart

Figure S2: Transitions from baseline to cancer, cardiovascular disease (CVD), type-2 diabetes (T2D) and specific multimorbidity patterns within the EPIC study

Figure S3: Subgroup analysis of associations between lifestly factors and cancer-cardiometabolic multimorbitiy

Figure S4: Absolute risk estimates for 55 years old men and women for values of the healthy lifestyle index

Figure S5: Sensitiviy analysis of associations between healthy lifestly index and cancer-cardiometabolic multimorbitiy

Figure S6: Sensitiviy analysis of associations between a simplified healthy lifestly indexe and cancer-cardiometabolic multimorbitiy

Figure S7: Absolute risk estimates for 65 years old men and women for values of a simplified healthy lifestyle index comparing individuals adhering to 4 healthy lifestlye habits vs. individuals adhereing to 1 healthy lifestyle habit.

Figure S8. Absolute risk estimateas for 65 years old men and women for values of the healthy lifestyle index to develop cancer-cardiometabolic multimorbodity after a less and more survivable cancer.

Table S1. Scoring of the healthy lifestyle index (HLI) and its simplified version

Modifiable lifestyle factor	Scoring HLI		Scoring sHLI	
Body mass index				
kg/m^2	≥22 to <24	4	≥18.5 to <25	1
	<22	3	≥25 to <30	0.5
	≥24 to <26	2	<18.5	0
	≥26 to <30	1	≥30	0
	≥30	0		
Smoking status				
Categories	Never	4	Never	1
	Former	2	Former	0.5
	Current	0	Current	0
Physical activity index[1]				
Categories	Active	4	Active	1
	Moderately active	3	Moderately active	1
	Moderately inactive	1	Moderately inactive	0
	Inactive	0	Inactive	0
Alcohol intake				
g/d	<6	4	<6 (W) or <12 (M)	1
	≥6 to <12	3	≥6 (W) or ≥12 (M)	0
	≥12 to <25	2		
	≥ to <60	1		
	>60	0		
Mediterranean diet score[2]				
Quintiles	Q5	4	≥ median	1
	Q4	3	< median	0
	Q3	2		
	Q2	1		
	Q1	0		
Range		0 to 20		0 to 5

HLI, healthy lifestyle index
sHLI, simplified healthy lifestyle index
W, women; M, men

[1] Wareham NJ, Jakes RW, Rennie KL, Schuit J, Mitchell J, Hennings S, Day NE. Validity and repeatability of a simple index derived from the short physical activity questionnaire used in the European Prospective Investigation into Cancer and Nutrition (EPIC) study. Public Health Nutr. 2003 Jun;6(4):407-13.

[2] Buckland G, González CA, Agudo A, Vilardell M, Berenguer A, Amiano P, et al. Adherence to the mediterranean diet and risk of coronary heart disease in the spanish EPIC cohort study. Am J Epidemiol. 2009;170(12):1518–29.

Figure S1. Flowchart of exclusion steps in the EPIC (European Prospective Investigation into Cancer and Nutrition) multimorbidity data.

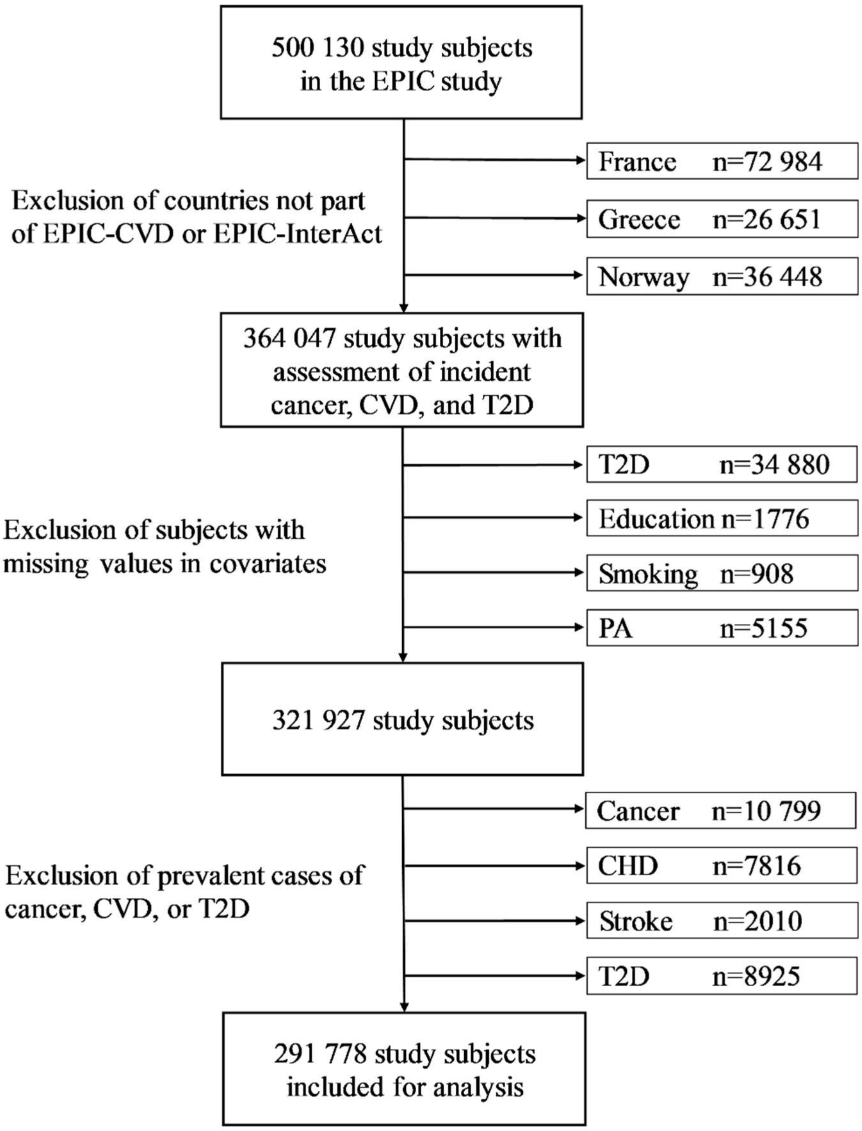

Figure S2. Transitions from baseline to cancer, cardiovascular disease (CVD), type-2 diabetes (T2D) and specific multimorbidity patterns within the EPIC study

State-specific number of events are in boxes, and transition-specific number of events and incidence rates per 1000 person-years are reported on arrows.

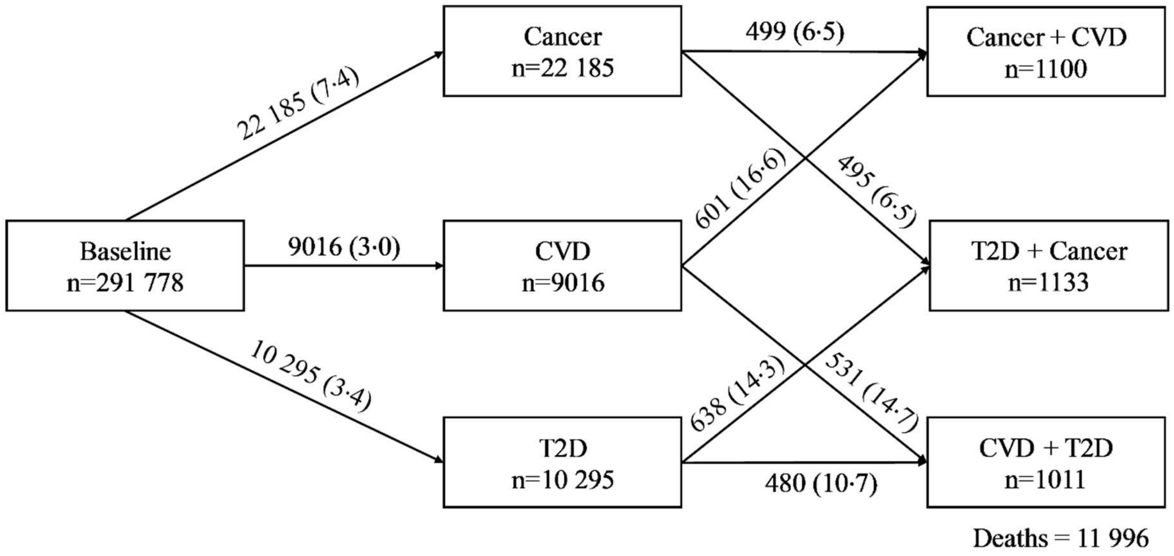

Figure S3. Subgroup analysis of associations of a 3-unit increment (~1 standard deviation) in the healthy lifestyle index (HLI) with first cancer at any site, cardiovascular disease (CVD), type-2 diabetes (T2D), and transitions to cancer-cardiometabolic multimorbidity.

Cox proportional hazard models, stratified by age at inclusion (1-year categories), sex, centre and transition, in a clock-forward multi-state analysis with age as primary time variable, adjusted for education level (no schooling, primary, secondary and university or more), height (continuous), an indicator of alcohol use (no/yes), total energy intake (kcal/day), and use of hormones and menopausal status in women.

Subgroups	Cases	PY	HR (95% CI)
**Women**			
Cancer	13 676	1 933 890	0.90 (0.88, 0.91)
CVD	3 788	1 933 890	0.75 (0.73, 0.78)
T2D	5 123	1 933 890	0.66 (0.65, 0.68)
Cancer → Multimorbidity	488	52 395	0.74 (0.67, 0.81)
CVD → Multimorbidity	395	15 357	0.90 (0.80, 1.02)
T2D → Multimorbidity	451	22 594	0.74 (0.66, 0.83)
**Men**			
Cancer	8 509	1 062 269	0.87 (0.85, 0.89)
CVD	5 228	1 062 269	0.75 (0.73, 0.77)
T2D	5 172	1 062 269	0.64 (0.62, 0.66)
Cancer → Multimorbidity	506	23 835	0.74 (0.67, 0.82)
CVD → Multimorbidity	737	20 773	0.78 (0.71, 0.86)
T2D → Multimorbidity	667	22 084	0.86 (0.78, 0.95)
**Age group 55 years and younger**			
Cancer	10 028	2 014 597	0.88 (0.86, 0.90)
CVD	3 394	2 014 597	0.70 (0.67, 0.72)
T2D	5 074	2 014 597	0.61 (0.60, 0.63)
Cancer → Multimorbidity	294	36 741	0.76 (0.66, 0.87)
CVD → Multimorbidity	350	13 866	0.87 (0.76, 1.00)
T2D → Multimorbidity	369	21 883	0.86 (0.75, 0.98)
**Age group older than 55 years**			
Cancer	12 157	981 562	0.89 (0.87, 0.90)
CVD	5 622	981 562	0.78 (0.76, 0.81)
T2D	5 221	981 562	0.68 (0.66, 0.70)
Cancer → Multimorbidity	700	39 489	0.72 (0.66, 0.79)
CVD → Multimorbidity	782	22 264	0.80 (0.74, 0.87)
T2D → Multimorbidity	749	22 795	0.79 (0.73, 0.87)
**Region 1 (Denmark, Sweden)**			
Cancer	9 003	943 560	0.88 (0.86, 0.90)
CVD	4 503	943 560	0.72 (0.70, 0.75)
T2D	5 004	943 560	0.64 (0.63, 0.66)
Cancer → Multimorbidity	580	32 303	0.70 (0.64, 0.77)
CVD → Multimorbidity	655	17 537	0.84 (0.77, 0.92)
T2D → Multimorbidity	575	19 412	0.78 (0.70, 0.86)
**Region 2 (Germany, the Netherlands, the UK)**			
Cancer	7 827	1 189 351	0.89 (0.87, 0.91)
CVD	2 524	1 189 351	0.76 (0.73, 0.80)
T2D	2 004	1 189 351	0.60 (0.58, 0.63)
Cancer → Multimorbidity	165	23 222	0.69 (0.56, 0.83)
CVD → Multimorbidity	216	10 301	0.67 (0.56, 0.81)
T2D → Multimorbidity	228	10 310	0.84 (0.70, 1.01)
**Region 3 (Italy, Spain)**			
Cancer	5 355	863 247	0.89 (0.86, 0.91)
CVD	1 989	863 247	0.80 (0.76, 0.83)
T2D	3 287	863 247	0.70 (0.67, 0.72)
Cancer → Multimorbidity	249	20 705	0.88 (0.76, 1.03)
CVD → Multimorbidity	261	8 292	0.88 (0.74, 1.06)
T2D → Multimorbidity	315	14 956	0.90 (0.78, 1.04)

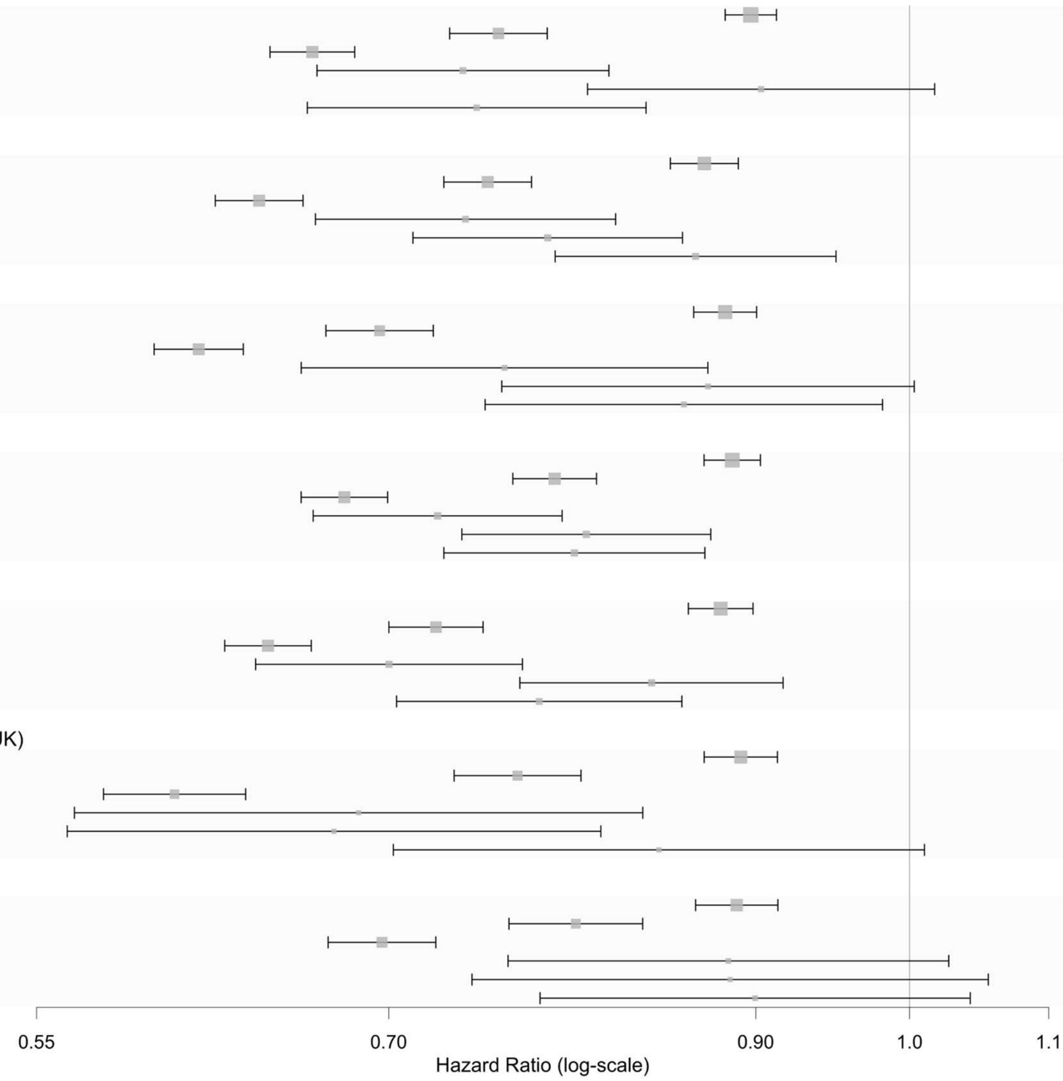

Figure S4. Cumulative incidence functions to develop first cancer at any site, cardiovascular disease (CVD), type-2 diabetes (T2D), and subsequent cancer-cardiometabolic multimorbodity (MM) for 55 years old men (dotted) and women (continuous) for values of the healthy lifestyle index (HLI) of 15 (healthy, 85th percentile in green) and 5 (unhealthy, 4th percentile in red); the HLI ranges from 0 to 20 units, with greater scores reflecting healthy lifesyles.

Cancer refers to first malignant tumors at any site excl. non-melanoma skin cancer. Deaths were censored and not modelled as a separate outcome.

The model was stratified for centre, sex, and adjusted for education level (no schooling, primary, secondary and university or more), height (continuous), binary indicator of alcohol use (no/yes), total energy intake (kcal/day), and use of hormones and menopausal status in women.

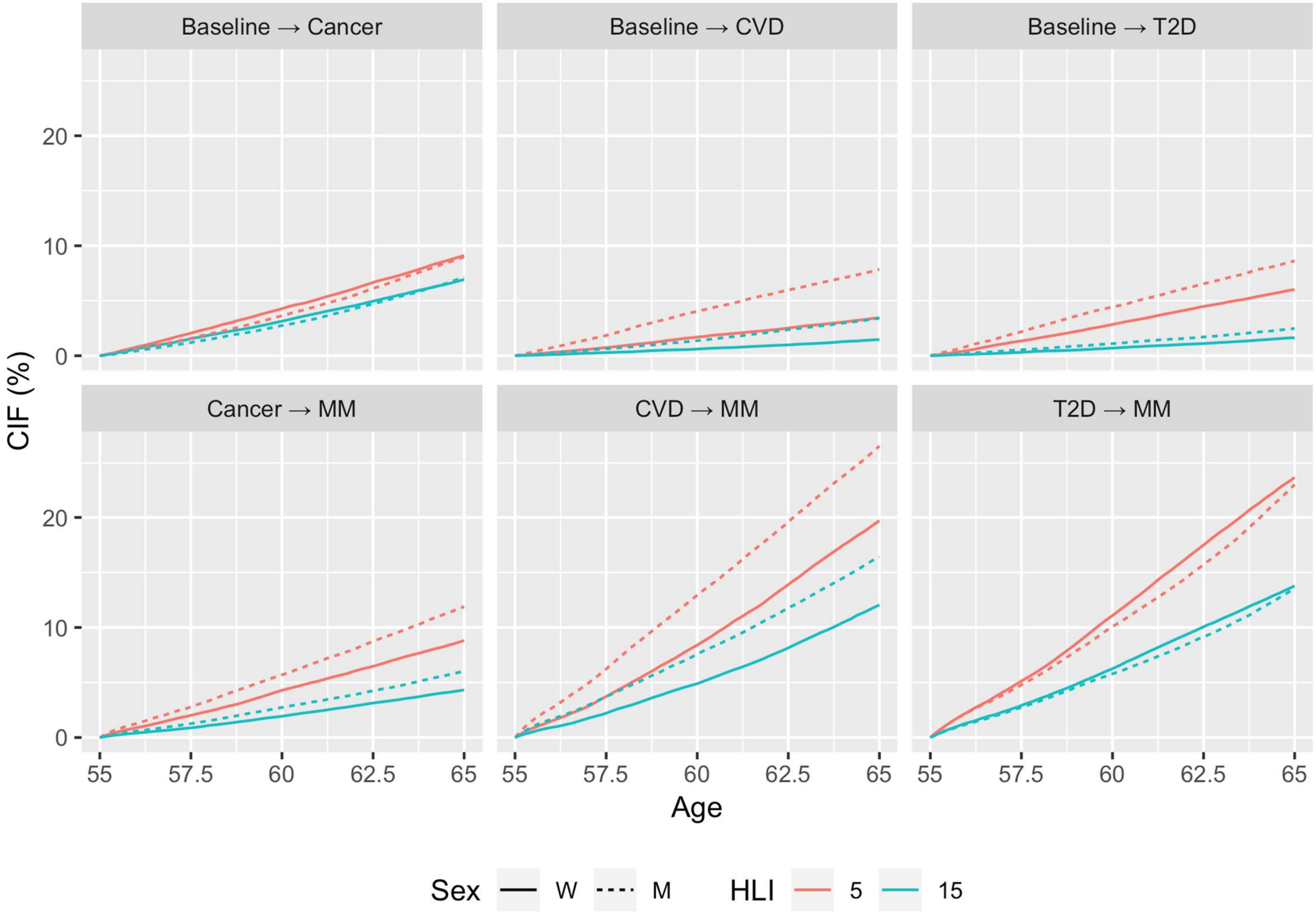

Figure S5. Sensitivity analysis excluding in turn one of the components of the healthy lifestyle index (HLI) of associations of a 3-unit increment (~1 standard deviation) in the HLI with first cancer at any site, cardiovascular disease (CVD), type-2 diabetes (T2D), and transitions to cancer-cardiometabolic multimorbidity.

Cox proportional hazard models, stratified by age at inclusion (1-year categories), sex, centre and transition, in a clock-forward multi-state analysis with age as primary time variable, adjusted for education level (no schooling, primary, secondary and university or more), height (continuous), an indicator of alcohol use (no/yes), total energy intake (kcal/day), and use of hormones and menopausal status in women.

Exposure variable	Cases	PY	HR (95% CI)
**HLI, per 1 SD**			
Cancer	22,185	2,996,159	0.89 (0.88, 0.91)
CVD	9,016	2,996,159	0.77 (0.75, 0.78)
T2D	10,295	2,996,159	0.67 (0.66, 0.69)
Cancer → Multimorbidity	994	101,261	0.75 (0.71, 0.81)
CVD → Multimorbidity	1,132	42,865	0.84 (0.79, 0.90)
T2D → Multimorbidity	1,118	54,648	0.82 (0.77, 0.88)
**BMI excluded, per 1 SD**			
Cancer	22,185	2,996,159	0.88 (0.87, 0.89)
CVD	9,016	2,996,159	0.79 (0.77, 0.80)
T2D	10,295	2,996,159	0.87 (0.85, 0.88)
Cancer → Multimorbidity	994	101,261	0.86 (0.81, 0.92)
CVD → Multimorbidity	1,132	42,865	0.90 (0.84, 0.95)
T2D → Multimorbidity	1,118	54,648	0.82 (0.77, 0.88)
**Smoking excluded, per 1 SD**			
Cancer	22,185	2,996,159	0.95 (0.94, 0.97)
CVD	9,016	2,996,159	0.93 (0.91, 0.95)
T2D	10,295	2,996,159	0.66 (0.65, 0.67)
Cancer → Multimorbidity	994	101,261	0.82 (0.77, 0.88)
CVD → Multimorbidity	1,132	42,865	0.88 (0.82, 0.94)
T2D → Multimorbidity	1,118	54,648	0.94 (0.88, 1.00)
**Physical activity excluded, per 1 SD**			
Cancer	22,185	2,996,159	0.88 (0.86, 0.89)
CVD	9,016	2,996,159	0.76 (0.75, 0.78)
T2D	10,295	2,996,159	0.66 (0.65, 0.68)
Cancer → Multimorbidity	994	101,261	0.73 (0.68, 0.79)
CVD → Multimorbidity	1,132	42,865	0.81 (0.75, 0.87)
T2D → Multimorbidity	1,118	54,648	0.82 (0.77, 0.89)
**Alcohol consumption excluded, per 1 SD**			
Cancer	22,185	2,996,159	0.90 (0.89, 0.91)
CVD	9,016	2,996,159	0.75 (0.74, 0.77)
T2D	10,295	2,996,159	0.63 (0.62, 0.64)
Cancer → Multimorbidity	994	101,261	0.75 (0.70, 0.80)
CVD → Multimorbidity	1,132	42,865	0.84 (0.78, 0.89)
T2D → Multimorbidity	1,118	54,648	0.84 (0.79, 0.89)
**Mediterranean diet excluded, per 1 SD**			
Cancer	22,185	2,996,159	0.90 (0.89, 0.91)
CVD	9,016	2,996,159	0.79 (0.78, 0.81)
T2D	10,295	2,996,159	0.67 (0.66, 0.69)
Cancer → Multimorbidity	994	101,261	0.80 (0.75, 0.86)
CVD → Multimorbidity	1,132	42,865	0.85 (0.80, 0.91)
T2D → Multimorbidity	1,118	54,648	0.84 (0.79, 0.90)

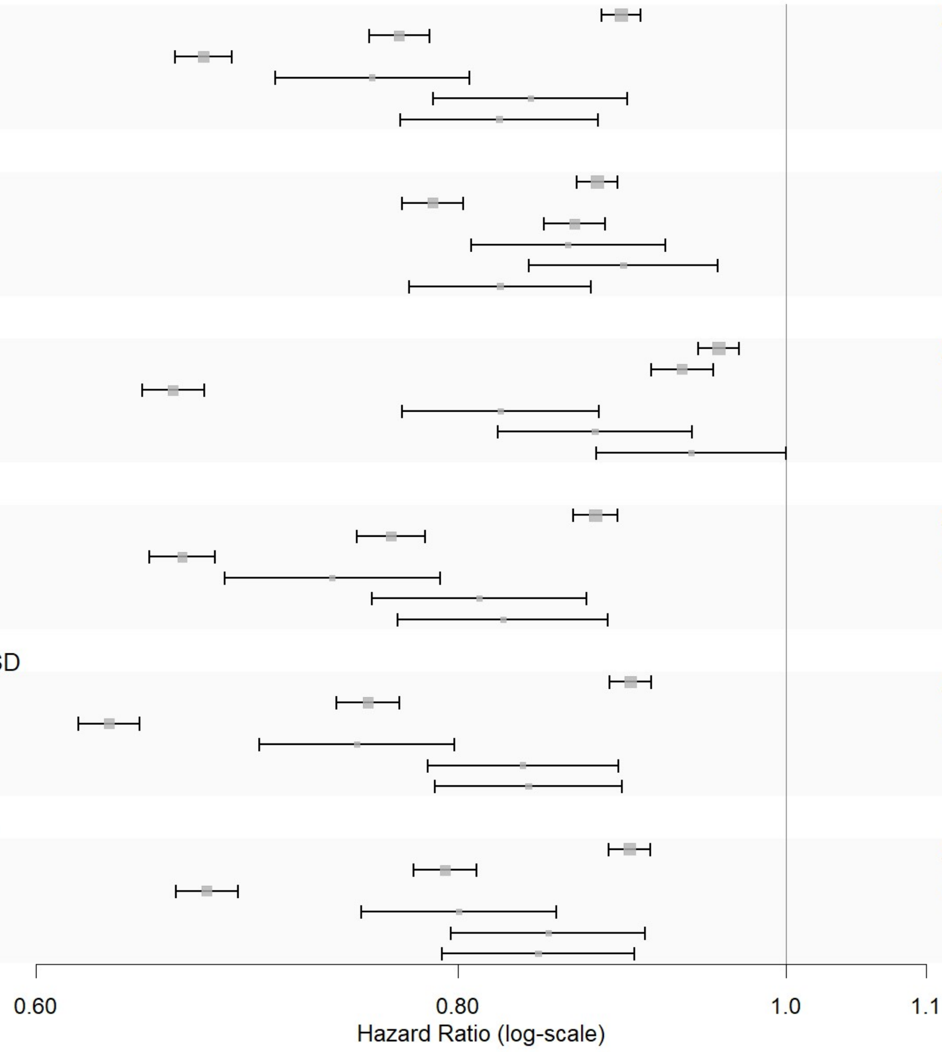

Figure S6. Sensitivity analysis comparing associations of 1 standard deviation (SD) increments in [A] the healthy lifestyle index (HLI), and [B] a simplified HLI, with first cancer at any site, cardiovascular disease (CVD), type-2 diabetes (T2D), and transitions to cancer-cardiometabolic multimorbidity.

Cox proportional hazard models, stratified by age at inclusion (1-year categories), sex, centre and transition, in a clock-forward multi-state analysis with age as primary time variable, adjusted for education level (no schooling, primary, secondary and university or more), height (continuous), an indicator of alcohol use (no/yes), total energy intake (kcal/day), and use of hormones and menopausal status in women.

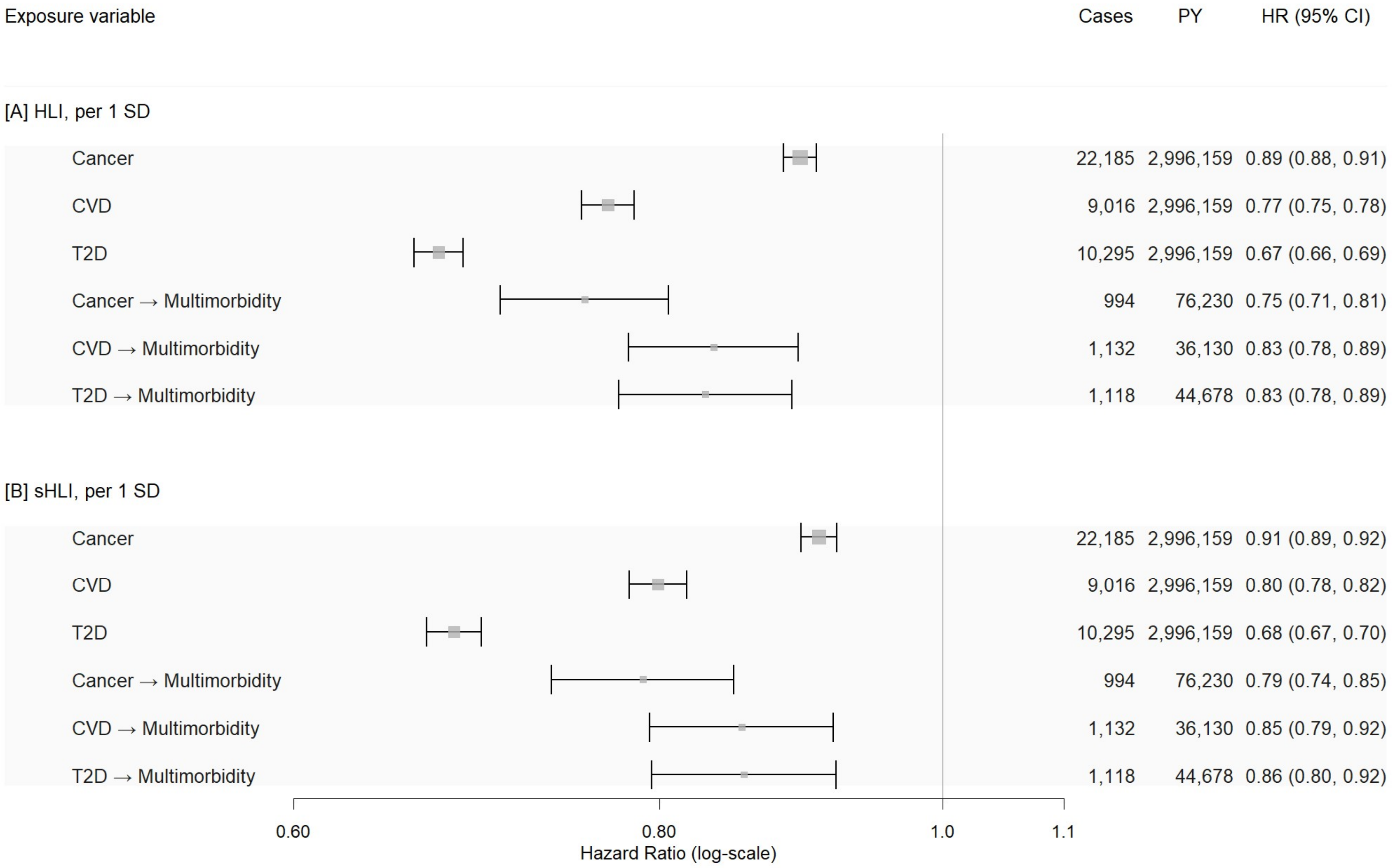

Figure S7. Cumulative incidence functions to develop first cancer at any site, cardiovascular disease (CVD), type-2 diabetes (T2D), and subsequent cancer-cardiometabolic multimorbodity (MM) for 65 years old men (dotted) and women (continuous) for values of the **simplified healthy lifestyle index (sHLI)** of 4 (four healthy lifestyle habits, 90th percentile in green) and 1 (unhealthy [one healthy lifestyle habit], 5th percentile in red); the sHLI ranges from 0 to 5 units.

Cancer refers to first malignant tumors at any site excl. non-melanoma skin cancer. Deaths were censored and not modelled as a separate outcome.

The model was stratified for centre, sex, and adjusted for education level (no schooling, primary, secondary and university or more), height (continuous), binary indicator of alcohol use (no/yes), total energy intake (kcal/day), and use of hormones and menopausal status in women

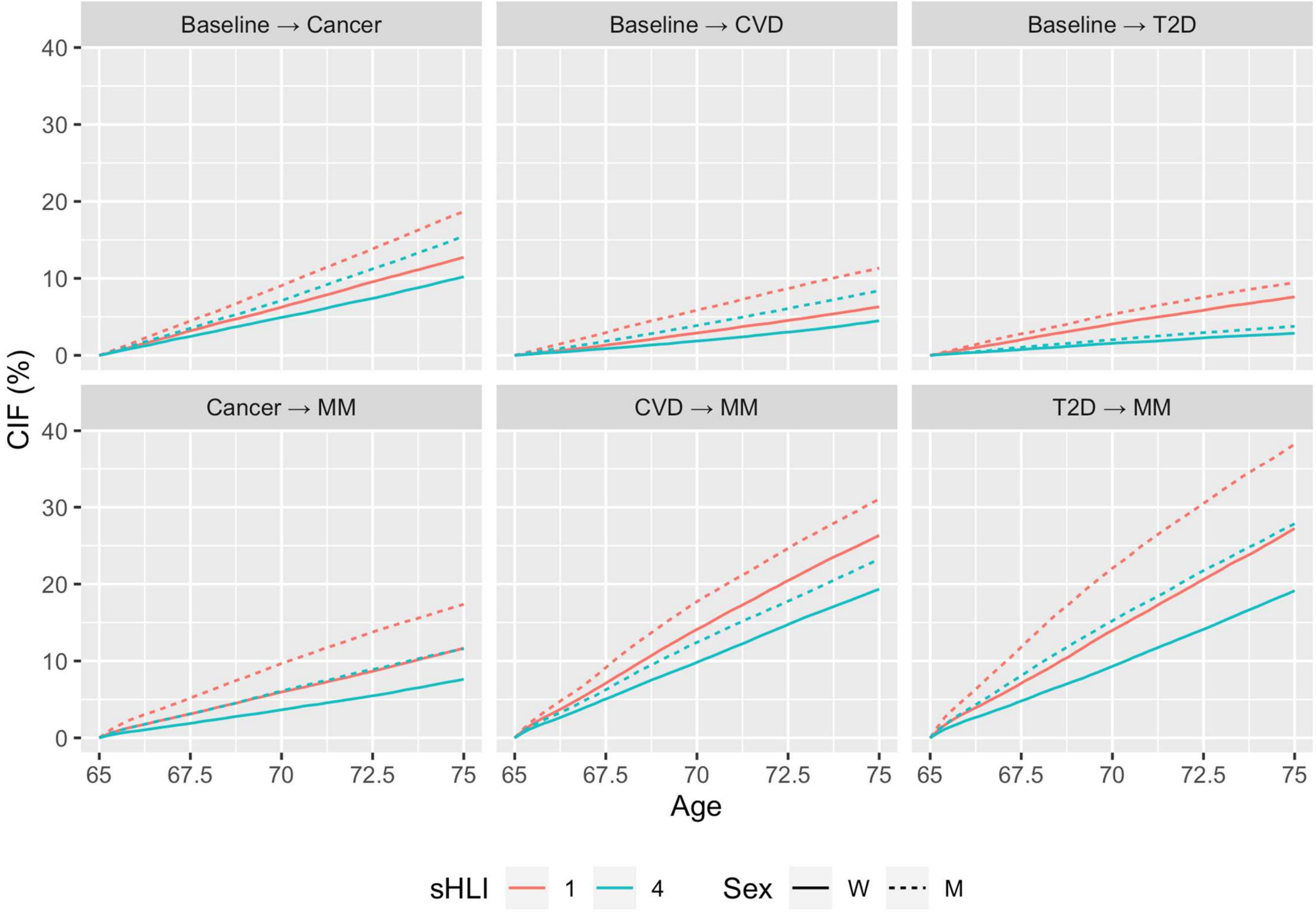

Figure S8. Cumulative incidence functions to develop cancer-cardiometabolic multimorbodity (MM) for 65 years old men (dotted) and women (continuous) after a less and more survivable cancer*, and after cardiovascular disease (CVD), and type-2 diabetes (T2D) for values of the healthy lifestyle index (HLI) of 15 (healthy, 85th percentile in green) and 5 (unhealthy, 4th percentile in red); the HLI ranges from 0 to 20 units, with greater scores reflecting healthy lifesyles.

*Cancer refers to first malignant tumors at any site excl. non-melanoma skin cancer, which were grouped according to 5-year relative survival rates into two distinct groups: (1) “less survivable cancer” with 5-year relative survival rates of less than 40% included pancreas, gallbladder, liver, lung, oesophagus, glioma, and stomach; (2) “more survivable cancer” with 5-year relative survival rates of equal or higher than 40% included ovarian, multiple myeloma, leukemia, colorectum, head and neck, non-Hodgkin’s lymphoma, kidney, meningioma, bladder, endometrium, breast, thyroid, and prostate.

Deaths were censored and not modelled as a separate outcome.

The model was stratified for centre, sex, and adjusted for education level (no schooling, primary, secondary and university or more), height (continuous), binary indicator of alcohol use (no/yes), total energy intake (kcal/day), and use of hormones and menopausal status in women

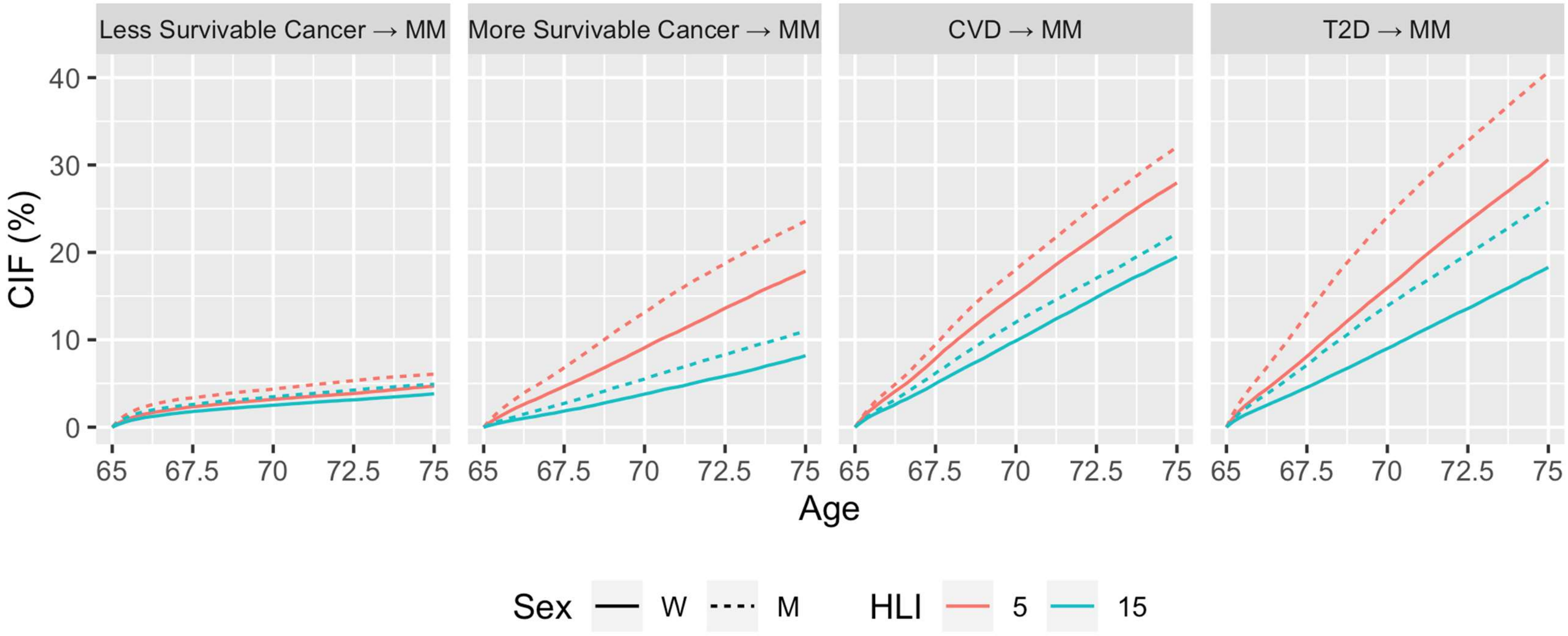

Supplement: Supplementary file 1 — Additional file 1: Figure S1. Provides the participant flow chart. Figure S2. Transitions to specific cancer and cardio-metabolic multimorbidity patterns. Figure S3. Results of subgroup analyses. Figure S4. CIFs for men and women of 55 years of age. Figure S5. Results of sensitivity analyses. Figure S6. Results for simplified HLI. Figure S7. CIFs for men and women of 55 years of age using the simplified HLI. and Figure S8. CIFs for men and women of 55 years of age for two groups of cancers. Table S1. Scoring of the healthy lifestyle index (HLI) and its simplified version. [file 12916_2019_1474_MOESM1_ESM.pdf]
